# Vitexin Alleviates Knee Osteoarthritis-Associated Cartilage Damage Associated with F3 Modulation

**DOI:** 10.3390/genes17091148

**Published:** 2026-09-19

**Authors:** Yanxiong Gu, Minshi Xiao, Ziyue Wu, Yunuo Shi, Wenhui Geng, Xingxiaoyu Lin, Xiaoqiang Yang

**Affiliations:** 1 State Key Laboratory of Traditional Chinese Medicine Syndrome/Guangzhou University of Chinese Medicine, Guangzhou 510405, China; 2 The Third Clinical Medical School, Guangzhou University of Chinese Medicine, Guangzhou 510405, China

**Keywords:** knee osteoarthritis, cartilage damage, F3

## Abstract

Background/Objects: Knee osteoarthritis (KOA) is characterized by pain and progressive cartilage damage, but the underlying molecular mechanisms remain incompletely understood. This study aimed to identify candidate genes through pain-related screening in KOA and explore the potential mechanism by which Vitexin alleviates cartilage damage. Methods: Transcriptomic analysis was performed on peripheral blood samples from control and KOA model mice, and differentially expressed genes were intersected with pain-related genes to identify candidate targets. GO enrichment analysis was subsequently performed to explore their potential biological functions. The role of F3 in KOA was further investigated in vivo using F3-targeting siRNA, followed by VEGFA rescue experiments to evaluate its association with CD31-positive vascular changes. Safranin O/Fast Green staining and immunofluorescence staining for F3, CD31, and COL2A1 were used to assess cartilage damage and angiogenesis. Finally, Vitexin was administered to KOA mice to investigate its potential effects on F3-associated pathological changes. Results: Integration of transcriptomic data with pain-related genes identified F3 and F8 as candidate genes in KOA. Exploratory GO analysis indicated that F3 was annotated to several angiogenesis-related biological processes. F3 protein expression was markedly increased in the tibial plateau of KOA mice. F3 knockdown reduced CD31 expression, restored COL2A1 expression and cartilage proteoglycan content, and attenuated cartilage damage. VEGFA administration reversed the F3 knockdown-induced reduction in CD31-positive vascular signals and the associated improvement in cartilage damage without restoring F3 expression, suggesting that vascular changes may represent a downstream process associated with F3. Furthermore, Vitexin treatment reduced F3 and CD31 expression, partially restored COL2A1 expression, and alleviated cartilage proteoglycan loss in KOA mice. Conclusions: F3 may contribute to KOA-associated cartilage damage by promoting pathological angiogenesis. Vitexin treatment reduced F3 expression and CD31-positive vascular signals, accompanied by alleviation of cartilage damage. These findings identify F3 as a candidate derived from pain-related gene screening and suggest its potential involvement in pathological angiogenesis and cartilage damage in KOA.

## 1. Introduction

Knee osteoarthritis (KOA) is a prevalent degenerative joint disease that affects hundreds of millions of individuals worldwide, with its incidence increasing substantially in the context of population aging and rising obesity rates [1,2,3]. Pain is one of the most prominent and debilitating manifestations of KOA and represents a major determinant of patients’ quality of life [4,5]. However, the molecular mechanisms underlying KOA-related pain remain incompletely understood, highlighting the need to identify novel molecular factors involved in the development and persistence of KOA pain.

Pain is a prominent clinical manifestation of KOA and is closely associated with disease burden and progression [6]. The onset and exacerbation of pain often accompany increased structural damage and inflammatory activity within the affected joint [7]. Given the close association between pain development and alterations in pain-related protein expression or activity, we hypothesized that identifying key pain-related proteins in KOA and investigating their functional roles may provide a novel perspective for understanding the molecular mechanisms underlying KOA.

Angiogenesis is an important pathological feature of KOA [8]. In the subchondral bone, aberrant neovascularization is often accompanied by the ingrowth of sensory nerve fibers and may further exacerbate local inflammation by promoting the release of pro-inflammatory mediators, thereby activating nociceptors and contributing to the development and progression of KOA pain [9]. Accordingly, suppressing aberrant subchondral angiogenesis may represent a potential strategy for alleviating KOA pain [10]. Based on these findings, we further hypothesized that candidate genes identified through pain-related screening may be associated with aberrant subchondral angiogenesis and KOA-associated cartilage damage.

Vitexin is a naturally occurring flavonoid widely distributed in various medicinal and edible plants. In addition to its analgesic properties, Vitexin has been reported to exhibit multiple biological activities, including anti-inflammatory and antioxidant effects [11]. Previous studies have also shown that Vitexin can attenuate inflammation and alleviate cartilage damage in KOA [12,13,14,15,16]. However, despite its established analgesic and chondroprotective effects, whether Vitexin modulates candidate pain-related molecular factors and thereby ameliorates KOA-associated pathological changes remains largely unexplored. Elucidating this potential mechanism may provide new insights into the therapeutic effects of Vitexin in KOA.

In this study, we integrated transcriptomic analysis with in vivo experiments to identify candidate genes through pain-related screening and investigate their potential roles in KOA. We first screened KOA-associated DEGs against a curated pain-related gene set and subsequently investigated the association of the identified candidate F3 with pathological angiogenesis and cartilage damage. We further investigated the effects of Vitexin on F3 expression and KOA-associated cartilage damage. These findings provide new insights into the potential molecular changes associated with the protective effects of Vitexin.

### Objectives

This study aimed to identify candidate genes through pain-related screening in KOA, investigate the role of F3 in pathological angiogenesis and cartilage damage, and explore the potential mechanism by which Vitexin exerts its protective effects.

## 2. Materials and Methods

### 2.1. Materials and Reagents

VEGF164 (HY-P7312), vitexin (HY-N0013), sodium iodoacetate (HY-D0849), (Asp-Ser-Ser)6 (HY-P11552) were obtained from MedChemExpress (MCE). Isoflurane (R510-22) was obtained from RWD Life Science. SevenFast RNAExtraction kit (SM130-02) was obtained from Seven. F3 (APC-FcA98114), CD31 (28083-1-AP) and Col2a1 (86139-2-RR) were obtained from Proteintech. 4% paraformaldehyde (BL539A) was obtained from Biosharp. Safranin O/Fast Green staining kit (S191031) was obtained from Pinuofei Biotechnology.

### 2.2. siRNA Construction

The F3-targeting siRNA was designed based on the mouse F3 reference sequence (NM_010171.3), with the targeting sequence GGAUAUCAACUGAUUUCAAGA. For in vivo delivery, F3 siRNA was loaded into a bone-targeting (Asp-Ser-Ser)_6_ [(DSS)_6_]-conjugated liposomal delivery system according to a previously reported method [17], with minor modifications. Briefly, liposomes were prepared using dipalmitoyl-sn-glycero-3-phosphocholine (DPPC), 1,2-distearoyl-sn-glycero-3-phosphoethanolamine-N-[maleimide(polyethylene glycol)-2000] (DSPE-PEG-maleimide), and cholesterol. The lipid components were dissolved in a chloroform/methanol mixture and subjected to vacuum evaporation to form a dry lipid film, which was subsequently hydrated with DEPC-treated water. The resulting lipid suspension was sonicated and extruded through a 200 nm polycarbonate membrane. For bone-targeted modification, the liposomes were incubated with (DSS)_6_ at a peptide-to-lipid molar ratio of 1:2 at 37 °C for 1 h, followed by dialysis. F3 siRNA was loaded into the hydrated lipids at a lipid-to-siRNA weight ratio of 50:1 using six consecutive freeze–thaw cycles between liquid nitrogen and a 37 °C water bath, followed by extrusion to obtain (DSS)_6_-liposome-F3 siRNA. The prepared (DSS)_6_-liposome-F3 siRNA was administered to mice via tail vein injection at an siRNA dose of 10 mg/kg once daily for 15 consecutive days. A corresponding negative-control siRNA formulation [(DSS)_6_-liposome-siNC] was prepared using the same procedure and administered under identical conditions. The efficiency of F3 knockdown in the tibial plateau was subsequently evaluated by immunofluorescence staining.

### 2.3. Animal Experiments

Thirty 8-week-old male C57BL/6 mice were used in this study. All animals were housed under SPF conditions with free access to standard chow and water. Following a 1-week acclimatization period, mice with body weights below the normal range were excluded from the study. The remaining 30 mice were randomly divided into six groups: the Control, Model, siNC, F3 siRNA, VEGFA + F3 siRNA, and Vitexin groups. Except for the Control group, KOA was induced by intra-articular injection of sodium iodoacetate (MIA; 10 mg/mL, 50 μL per knee). After a 7-day induction period following MIA injection, the corresponding interventions were initiated and continued for 15 consecutive days [18]. The Control group received an equal volume of sterile saline by intra-articular injection. Following KOA induction, mice in the F3 siRNA group received F3 siRNA treatment. Mice in the VEGFA + F3 siRNA group received both F3 siRNA and VEGFA treatment, whereas mice in the Vitexin group received Vitexin treatment. VEGFA was administered by intraperitoneal injection at a dose of 0.5 μg/kg every 2 days, and Vitexin was administered by intraperitoneal injection at a dose of 15 mg/kg once daily for 15 consecutive days. All interventions were continued for 15 days. All animal procedures and experimental protocols were conducted in accordance with the guidelines approved by the Animal Ethics Committee of Guangzhou University of Chinese Medicine (Approval No. 20250717002). At the end of the intervention period, blood samples were collected from the retro-orbital venous plexus under deep isoflurane anesthesia. The hind limbs were subsequently harvested and stored at −80 °C until further analysis.

### 2.4. Transcriptomic Analysis

Three peripheral blood samples were collected from each group (Control and KOA groups) for transcriptomic sequencing, with each sample obtained independently from an individual animal. Total RNA was extracted from the peripheral blood samples, and RNA concentration, purity, and integrity were assessed to ensure that only qualified RNA samples were used for subsequent library preparation. Sequencing libraries were constructed from qualified RNA samples, followed by library quality assessment. The qualified libraries were subsequently subjected to high-throughput sequencing. After sequencing, the resulting raw reads were subjected to quality control, including the removal of adapter-containing reads and low-quality reads, to obtain high-quality clean reads for subsequent bioinformatics analyses. All six samples passed quality control and were retained for subsequent analyses, with no samples excluded.

### 2.5. Bioinformatics Analysis

Gene expression levels were quantified as fragments per kilobase of transcript per million mapped reads (FPKM). To minimize the potential influence of lowly expressed genes on downstream analyses, genes with FPKM > 1 in at least three of the six samples were retained. The filtered expression values were log_2_-transformed [log_2_(FPKM + 1)] and subsequently subjected to quantile normalization using the normalizeBetweenArrays function in the limma package. Based on the normalized expression matrix, differential expression analysis between the Control and KOA groups was performed using the limma package in R (version 4.3.1). *p* values were adjusted for multiple testing using the Benjamini–Hochberg method, and genes with |log_2_FC| > 1 and an adjusted *p* value < 0.05 were considered differentially expressed genes (DEGs) [19]. In addition, the GeneCards database was searched using the keyword “pain” on 3 July 2025, and the top 500 pain-related genes ranked by GeneCards relevance score were retrieved. The identified DEGs were then intersected with the pain-related gene set to identify candidate genes. Gene Ontology (GO) enrichment analysis of the overlapping genes was subsequently performed using the clusterProfiler package [20,21,22]. This analysis was considered exploratory and was used only to provide preliminary functional context rather than to establish statistically robust pathway-level enrichment.

### 2.6. Safranin O/Fast Green Staining

Safranin O/Fast Green staining. Paraffin-embedded bone tissue sections were deparaffinized in xylene and rehydrated with ethanol, followed by staining using a Safranin O/Fast Green staining kit. Sections were stained with Fast Green solution for 5 min, rinsed with distilled water, and subsequently stained with Safranin O solution for 2 min. The sections were then dehydrated with absolute ethanol, cleared in xylene, and mounted with neutral resin. Stained sections were examined and imaged under a microscope. For quantitative analysis, the Safranin O-positive area was measured using ImageJ software (v.1.51k). The mean value of the Control group was then used for normalization, and the value for each sample was divided by the mean value of the Control group to obtain the relative Safranin O-positive area.

### 2.7. Immunofluorescence Staining

Tissue sections were deparaffinized, rehydrated, and subjected to Tris-EDTA-mediated antigen retrieval. After blocking endogenous peroxidase activity with 3% H_2_O_2_. and nonspecific binding with 10% goat serum, sections were incubated with the indicated primary antibodies. For F3 immunofluorescence staining, sections were incubated with an anti-F3 primary antibody, followed by the corresponding secondary antibody. For CD31/Col2a1 double immunofluorescence staining, sections were sequentially incubated with anti-CD31 and anti-Col2a1 primary antibodies and the corresponding secondary antibodies, with TYR-488 and TYR-555 TSA used for signal amplification, respectively. Nuclei were counterstained with DAPI, and sections were mounted with antifade medium and stored at 4 °C in the dark. For quantitative analysis, three randomly selected fields from the tibial plateau were analyzed for each mouse under identical imaging conditions. Fluorescence intensity was quantified using ImageJ software. The values obtained from the three fields were averaged to generate a single value for each mouse. Each mouse was considered an independent biological replicate (*n* = 5 mice per group) for subsequent statistical analysis, and individual microscopic fields were not treated as independent replicates.

### 2.8. Statistical Analysis

Bioinformatics analyses were performed using R software (v4.3.1), while statistical analyses were conducted using SPSS (v24.0). Normality and homogeneity of variance were assessed using the Shapiro–Wilk and Brown–Forsythe tests, respectively. For comparisons between two groups, an unpaired Student’s *t*-test was used for normally distributed data with homogeneous variances, Welch’s unpaired *t*-test for normally distributed data with unequal variances, and the Mann–Whitney U test for non-normally distributed data. For comparisons among multiple groups, one-way ANOVA followed by Dunnett’s multiple-comparisons test was used for normally distributed data with homogeneous variances; Welch’s ANOVA followed by Dunnett’s T3 multiple-comparisons test was used when variances were unequal; and the Kruskal–Wallis test followed by Dunn’s multiple-comparisons test was used for non-normally distributed data. *p* value < 0.05 was considered statistically significant.

## 3. Results

### 3.1. Identification of F3 Through Pain-Related Gene Screening in KOA

**Figure 1:**
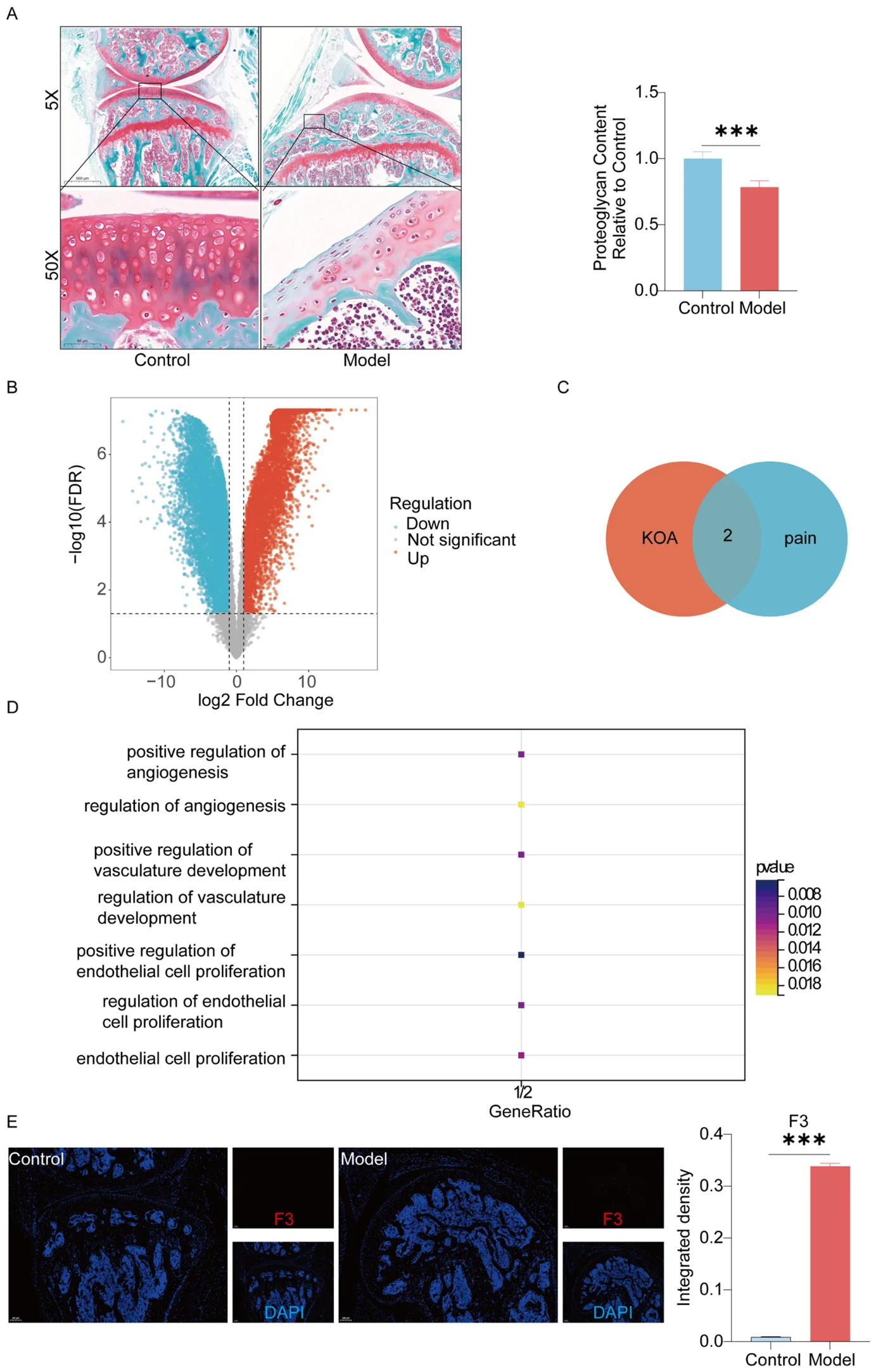
F3 was identified through pain-related gene screening in KOA. (**A**) Representative Safranin O/Fast Green staining images of cartilage morphology in the Control and Model groups, with corresponding magnified views. Scale bars = 500 μm (5X magnification, low-power overall views) and 50 μm (50X magnification, high-power magnified views). (**B**) Volcano plot showing differentially expressed genes (DEGs) between the Control and Model groups. (**C**) Venn diagram illustrating the intersection between DEGs and pain-related genes. (**D**) Dot plot showing exploratory Gene Ontology (GO) analysis of the two overlapping candidate genes, F3 and F8. (**E**) Representative immunofluorescence staining images showing F3 protein expression in the Control and Model groups. Red fluorescence indicates F3 protein, and blue fluorescence indicates cell nuclei stained with DAPI (4X, scale bar = 250 μm). *** *p* < 0.001.

To identify candidate genes potentially associated with pain-related molecular features in KOA, we first established a mouse model of KOA. Safranin O/Fast Green staining revealed marked cartilage matrix degradation at the tibial plateau in KOA mice (Figure 1A). We subsequently performed transcriptomic analysis of peripheral blood samples from the Control and KOA groups. Following quality control (Figure S1), a total of 13,476 differentially expressed genes (DEGs) were identified, including 6044 upregulated and 7432 downregulated genes (Figure 1B). These DEGs were further intersected with the top 500 pain-related protein-coding genes ranked by relevance in the GeneCards database, yielding two overlapping genes, F3 and F8 (Figure 1C) [23]. Exploratory GO analysis of the two overlapping genes (F3 and F8) identified several angiogenesis-related biological processes involving F3, including regulation of angiogenesis, vasculature development, and endothelial cell proliferation (Figure 1D). Given the limited number of input genes, these results were interpreted only as preliminary functional clues and were used to guide subsequent experimental investigation rather than as robust evidence of pathway-level enrichment. Consistent with the transcriptomic findings, immunofluorescence staining demonstrated significantly increased F3 protein expression in the tibial plateau cartilage matrix of the KOA group compared with the Control group (Figure 1E). Collectively, these findings identified F3 as a candidate gene through the intersection of KOA-associated DEGs and pain-related genes and suggested its potential involvement in angiogenesis-related biological processes, providing a basis for further mechanistic investigation.

### 3.2. F3 Knockdown Reduces CD31-Positive Vascular Signals and Cartilage Damage in KOA

**Figure 2:**
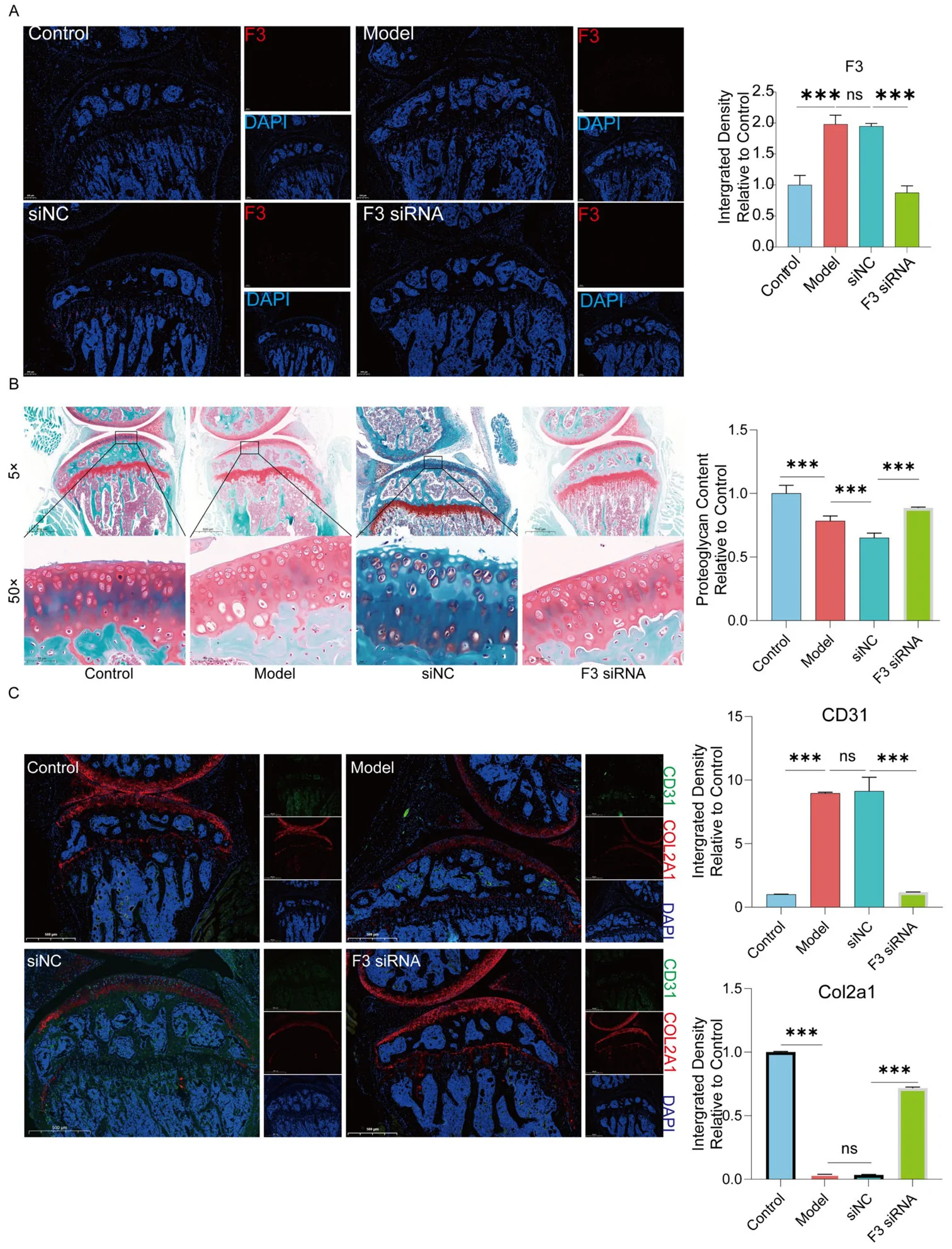
F3 Knockdown Reduces CD31-Positive Vascular Signals and Cartilage Damage in KOA. (**A**) Evaluation of F3 protein expression in the Model group following intervention with F3 siRNA and vector (magnification, 5×; scale bars = 200 µm). (**B**) Safranin O/Fast Green staining results for Control, Model, and F3 siRNA groups (overall view: magnification, 5×; scale bars = 500 µm; local view: magnification, 50×; scale bars = 50 µm). (**C**) Double immunofluorescence staining of CD31 and COL2A1 in the tibial plateau, with green representing CD31, red representing COL2A1, and blue representing DAPI (magnification, 5×; scale bars = 500 µm). not significant (ns) = *p* > 0.05, *** *p* < 0.001.

To evaluate whether F3 siRNA could effectively reduce F3 protein expression, the research team treated model mice with F3 siRNA and siNC. Results showed that the fluorescence intensity of F3 was significantly increased in the Model group, with no significant difference between the si-NC and Model groups, whereas F3 siRNA significantly reduced F3 expression in the tibial plateau (Figure 2A). Furthermore, Safranin O/Fast Green staining results indicated that the reduction in F3 protein expression significantly restored proteoglycan content in the tibial plateau (Figure 2B). Furthermore, the reduction in F3 protein expression led to decreased CD31 expression and a recovery of COL2A1 expression in the tibial plateau (Figure 2C). In summary, in this section, the research team applied F3 siRNA to evaluate the effect of reducing F3 expression on KOA.

### 3.3. VEGFA Reverses the Effects of F3 Knockdown on CD31-Positive Vascular Changes

**Figure 3:**
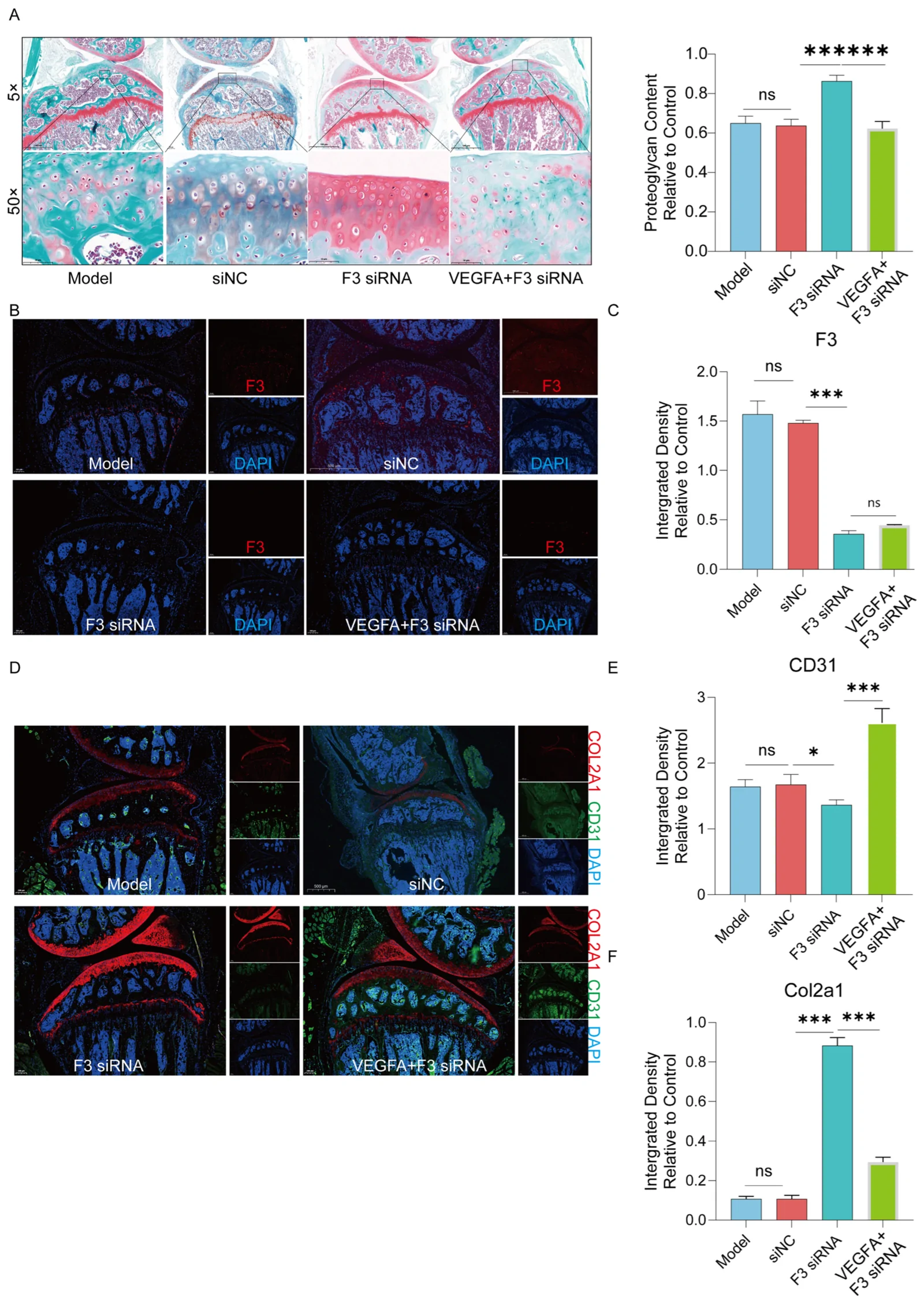
VEGFA Reverses the Effects of F3 Knockdown on CD31-Positive Vascular Changes. (**A**) Representative images of Safranin O/Fast Green staining of the tibial plateau in the Model, F3 siRNA, and F3 siRNA + VEGFA groups (overall view: magnification, 5×; scale bar = 500 µm; magnified view: magnification, 50×; scale bar = 50 µm). (**B**,**C**) Representative immunofluorescence images (**B**) and quantitative analysis of fluorescence intensity (**C**) for F3 protein in the tibial plateau across groups (magnification, 5×; scale bar = 200 µm). (**D**–**F**) Representative images (**D**) and double-immunofluorescence staining in the tibial plateau (magnification, 5×; scale bar = 200 µm), (**E**) quantitative evaluation of fluorescence intensity for CD31 (**F**) and COL2A1. not significant (ns) = *p* > 0.05, * *p* < 0.05, *** *p* < 0.001.

To further elucidate how F3 promotes subchondral angiogenesis, the research team conducted a rescue experiment by combining VEGFA treatment with F3 siRNA administration. Safranin O/Fast Green staining revealed that additional VEGFA treatment markedly reduced the proteoglycan content in the tibial plateau cartilage, indicating that VEGFA significantly reversed the protective effect of F3 siRNA on proteoglycan preservation (Figure 3A). However, immunofluorescence staining demonstrated that VEGFA intervention did not alter the reduced expression level of F3 protein induced by F3 siRNA (Figure 3B,C). Furthermore, VEGFA administration led to an elevation in the expression of the vascular endothelial marker CD31 and a concomitant decrease in the cartilage marker COL2A1 (Figure 3D–F). These findings suggest that VEGFA-mediated CD31-positive vascular changes may contribute to the downstream effects associated with F3 and cartilage damage.

### 3.4. Vitexin Reduces F3 Expression and CD31-Positive Vascular Signals in KOA

**Figure 4:**
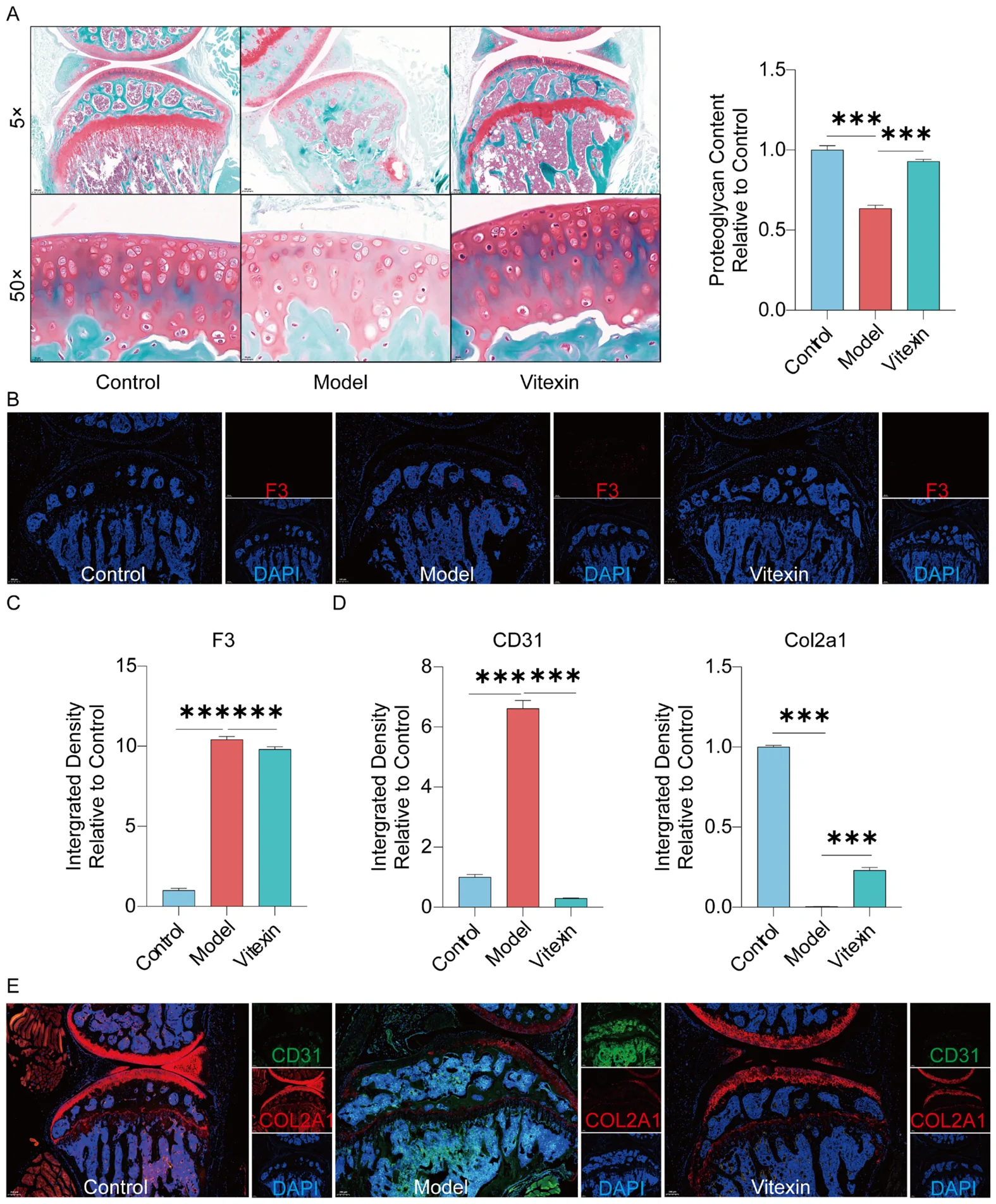
Vitexin Reduces F3 Expression and CD31-Positive Vascular Signals in KOA. (**A**) Safranin O/Fast Green staining showing the restoration of proteoglycan content in the tibial plateau after Vitexin treatment (overall view: 5×, scale bar = 500 μm; magnified view: 50×, scale bar = 50 μm). (**B**,**C**) Immunofluorescence staining and quantification of F3 expression in the tibial plateau (5×, scale bar = 200 μm). (**D**,**E**) Double immunofluorescence staining and quantification of CD31 and Col2a1 fluorescence intensity in the tibial plateau (5×, scale bar = 200 μm). *** *p* < 0.001.

To further investigate the effect of Vitexin on cartilage matrix degradation in KOA, KOA models were treated with Vitexin. Safranin O/Fast Green staining showed that Vitexin treatment significantly restored the proteoglycan content of the tibial plateau cartilage compared with the Model group (Figure 4A). We next explored the potential mechanism underlying this effect. Immunofluorescence staining revealed that F3 protein expression was markedly increased in the tibial plateau of the Model group, whereas Vitexin treatment significantly reversed this abnormal elevation (Figure 4B,C). Further double immunofluorescence staining showed that Vitexin treatment reduced CD31 expression while partially restoring Col2a1 expression (Figure 4D,E). Collectively, these findings indicate that Vitexin treatment reduces F3 expression and CD31-positive vascular signals in the subchondral region, accompanied by alleviation of proteoglycan loss in the tibial plateau cartilage of KOA mice.

## 4. Discussion

KOA-related pain severely affects patients’ quality of life and mobility [24]. In this study, we integrated transcriptomic data with a pain-related gene set to identify candidate genes in KOA and further investigated their potential pathological roles in vivo. F3 was subsequently selected for mechanistic investigation, with particular emphasis on its association with pathological angiogenesis and cartilage damage. Based on these findings, we further explored the potential mechanism by which Vitexin alleviates KOA-associated cartilage damage.

By intersecting KOA-associated DEGs with the pain-related gene set, we identified two candidate genes, F3 and F8. Previous studies have shown that F8 is primarily associated with pain in patients with hemophilia [25,26]. F8 deficiency impairs hemostasis following trauma, which may contribute to joint damage and chronic pain in these patients [27]. In contrast, F3 binds to circulating factor VIIa and subsequently cleaves and activates PAR1 at nerve endings, triggering downstream signaling pathways that ultimately induce pain [28,29,30,31]. In addition to its potential involvement in PAR1-related signaling, F3 (tissue factor) is a key initiator of the coagulation cascade. Increased tissue factor activity may promote local coagulation and fibrin deposition within or around the neovasculature, potentially contributing to a pro-inflammatory joint microenvironment and thereby facilitating inflammatory pain. This mechanism may provide an additional potential link between F3 and KOA-associated pain. However, because neither pain-related outcomes nor inflammatory markers were directly evaluated in the present study, this possibility remains speculative and requires further experimental validation. Our results showed that F3 protein expression was increased in KOA model mice, consistent with previous findings.

Exploratory GO analysis of the two candidate genes indicated that F3 was annotated to several angiogenesis-related biological processes. Given that only two genes were included in this analysis, these findings should be interpreted cautiously and were considered hypothesis-generating rather than definitive evidence of pathway enrichment. Based on this preliminary functional clue, we subsequently investigated the relationship between F3 and vascular changes in vivo. To further investigate the potential mechanisms associated with F3, we treated KOA model mice with F3-targeting siRNA. F3 siRNA treatment reduced F3 protein expression and alleviated cartilage damage in KOA model mice, suggesting that downregulation of F3 may contribute to the attenuation of cartilage injury. Given that abnormal angiogenesis is an important contributor to cartilage damage in KOA, we further examined the expression of CD31 and Col2a1. The results showed increased subchondral angiogenesis and decreased Col2a1 expression in the tibial plateau of KOA model mice. Following F3 siRNA treatment, F3 protein expression and CD31 expression were reduced, whereas Col2a1 expression was restored. These findings suggest that F3 upregulation may promote abnormal subchondral angiogenesis in the tibial plateau, thereby exacerbating cartilage damage and contributing to KOA progression. To further clarify the relationship between abnormal subchondral angiogenesis in the tibial plateau and F3 protein expression in KOA, we further investigated the effects of VEGFA, a protein that directly promotes angiogenesis [32]. Our results showed that VEGFA administration did not restore F3 protein expression in KOA model mice treated with F3 siRNA, whereas VEGFA reversed the reduction in CD31-positive vascular signals induced by F3 siRNA. These findings suggest that F3 may be associated with CD31-positive vascular changes in KOA. Collectively, these results suggest that F3 may contribute to CD31-positive vascular changes, which may in turn be associated with cartilage damage and KOA progression.

Based on the above findings, we next investigated whether pharmacological modulation of F3 might influence KOA-associated pathological changes. Vitexin has previously been reported to exert analgesic and chondroprotective effects; however, the molecular mechanisms underlying these effects remain incompletely understood. In the present study, Vitexin treatment reduced F3 and CD31 expression in the tibial plateau while partially restoring COL2A1 expression and cartilage proteoglycan content. These findings suggest that suppression of F3-associated CD31-positive vascular changes may represent a potential mechanism associated with the chondroprotective effect of Vitexin in KOA. However, because pain-related outcomes were not directly assessed, whether modulation of F3 contributes to the analgesic effects of Vitexin requires further investigation.

Compared with previous studies, our study investigated KOA-associated molecular alterations by integrating pain-related gene screening with experimental analysis of abnormal angiogenesis and cartilage damage, providing further insights into KOA pathogenesis [33,34]. On this basis, we preliminarily explored the potential mechanism by which vitexin alleviates KOA cartilage damage.

Nevertheless, this study has several limitations. Most importantly, although F3 was identified by intersecting KOA-associated DEGs with pain-related genes, pain-related behavioral outcomes were not directly assessed in the present study. Therefore, our findings do not establish a direct relationship between F3 expression and KOA pain, nor do they demonstrate that F3 downregulation or Vitexin treatment alleviates pain. Future studies incorporating validated pain-related behavioral assessments, together with clinical specimens, are required to clarify the relationship between F3 and KOA-associated pain. In addition, the present study does not establish that the protective effects of Vitexin are directly mediated by F3. Further rescue experiments, particularly F3 overexpression combined with Vitexin treatment, are required to determine whether the effects of Vitexin are F3-dependent. In addition, angiogenesis was primarily evaluated based on CD31 immunofluorescence in the present study. Therefore, these findings should be interpreted as CD31-positive vascular changes rather than definitive evidence of pathological angiogenesis. Further studies using additional vascular markers and morphological assessments are required to characterize pathological angiogenesis more comprehensively.

In conclusion, this study identified F3 as a candidate gene through the intersection of KOA-associated DEGs and pain-related genes. Subsequent in vivo experiments suggest that F3 may contribute to KOA-associated cartilage damage and may be associated with increased CD31-positive vascular changes. VEGFA rescue experiments further support vascular changes as a potential downstream process associated with F3. Vitexin reduced F3 expression and CD31-positive vascular signals and alleviated cartilage matrix damage, suggesting a potential F3-associated mechanism underlying its chondroprotective effects. The relationship between F3 and KOA-associated pain remains to be directly established in future studies.

## Data Availability

The datasets used and/or analyzed during the current study are available from the corresponding author on reasonable request.

## Supplementary Materials

The following supporting information can be downloaded at: https://www.mdpi.com/article/10.3390/genes17091148/s1, Figure S1: Quality assessment of transcriptome results.

## Abbreviations

The following abbreviations are used in this manuscript:

KOA	Knee Osteoarthritis
MIA	Monosodium Iodoacetate
DEGs	Differentially Expressed Genes
GO	Gene Ontology
SPF	Specific Pathogen-Free
DAPI	4′,6-Diamidino-2-Phenylindole
TSA	Tyramide Signal Amplification
